# Distinct transcriptome profiles identified in normal human bronchial epithelial cells after exposure to γ-rays and different elemental particles of high Z and energy

**DOI:** 10.1186/1471-2164-14-372

**Published:** 2013-06-01

**Authors:** Liang-Hao Ding, Seongmi Park, Michael Peyton, Luc Girard, Yang Xie, John D Minna, Michael D Story

**Affiliations:** 1 Departments of Radiation Oncology, University of Texas Southwestern Medical Center, 6000 Harry Hines Blvd, Dallas, TX 75390, USA; 2 The Hamon Center for Therapeutic Oncology Research, University of Texas Southwestern Medical Center, 6000 Harry Hines Blvd, Dallas, TX 75390, USA; 3 Pharmacology, University of Texas Southwestern Medical Center, 6000 Harry Hines Blvd, Dallas, TX 75390, USA; 4 Clinical Sciences, University of Texas Southwestern Medical Center, 6000 Harry Hines Blvd, Dallas, TX 75390, USA; 5 Simmons Comprehensive Cancer Center, University of Texas Southwestern Medical Center, 6000 Harry Hines Blvd, Dallas, TX 75390, USA; 6 Internal Medicine, University of Texas Southwestern Medical Center, 6000 Harry Hines Blvd, Dallas, TX 75390, USA

**Keywords:** Gene expression, HZE particles, Ionizing radiation, Human bronchial epithelial cells

## Abstract

**Background:**

Ionizing radiation composed of accelerated ions of high atomic number (Z) and energy (HZE) deposits energy and creates damage in cells in a discrete manner as compared to the random deposition of energy and damage seen with low energy radiations such as γ- or x-rays. Such radiations can be highly effective at cell killing, transformation, and oncogenesis, all of which are concerns for the manned space program and for the burgeoning field of HZE particle radiotherapy for cancer. Furthermore, there are differences in the extent to which cells or tissues respond to such exposures that may be unrelated to absorbed dose. Therefore, we asked whether the energy deposition patterns produced by different radiation types would cause different molecular responses. We performed transcriptome profiling using human bronchial epithelial cells (HBECs) after exposure to γ-rays and to two different HZE particles (^28^Si and ^56^Fe) with different energy transfer properties to characterize the molecular response to HZE particles and γ-rays as a function of dose, energy deposition pattern, and time post-irradiation.

**Results:**

Clonogenic assay indicated that the relative biological effectiveness (RBE) for ^56^Fe was 3.91 and for ^28^Si was 1.38 at 34% cell survival. Unsupervised clustering analysis of gene expression segregated samples according to the radiation species followed by the time after irradiation, whereas dose was not a significant parameter for segregation of radiation response. While a subset of genes associated with p53-signaling, such as *CDKN1A*, *TRIM22* and *BTG2* showed very similar responses to all radiation qualities, distinct expression changes were associated with the different radiation species. Gene enrichment analysis categorized the differentially expressed genes into functional groups related to cell death and cell cycle regulation for all radiation types, while gene pathway analysis revealed that the pro-inflammatory Acute Phase Response Signaling was specifically induced after HZE particle irradiation. A 73 gene signature capable of predicting with 96% accuracy the radiation species to which cells were exposed, was developed.

**Conclusions:**

These data suggest that the molecular response to the radiation species used here is a function of the energy deposition characteristics of the radiation species. This novel molecular response to HZE particles may have implications for radiotherapy including particle selection for therapy and risk for second cancers, risk for cancers from diagnostic radiation exposures, as well as NASA’s efforts to develop more accurate lung cancer risk estimates for astronaut safety. Lastly, irrespective of the source of radiation, the gene expression changes observed set the stage for functional studies of initiation or progression of radiation-induced lung carcinogenesis.

## Background

Exposure to galactic cosmic radiation (GCR) is considered a major health risk for astronauts [1-3] and is potentially mission compromising. GCR includes charged particles of high atomic number (Z) and energy (HZE). HZE particles can produce dense ionizations along their trajectory, which is unlike the random deposition of energy associated with electromagnetic radiations such as γ- or x-rays. The energy deposition of radiation is measured by the linear energy transfer (LET, keV/μ) which will vary based upon the particle atomic number (Z) and the kinetic energy of the particle. The density of such ionizations is variable and is often referred to as the particle quality. As the density of ionization increases it is thought to cause ever more complex and often irreparable DNA damage [4-6]. As a result HZE particles are more effective than low-LET radiation like γ- or x-rays for cell killing and mutation induction [7-14] and particle quality differences have also been shown to have varied biological effectiveness [15,16].

The current model for estimation of the health risks for HZE particle exposure is extrapolated from low-LET radiation data because of the lack of biological data for high-LET radiation. The uncertainties of the model due to possible qualitative differences between high- and low-LET radiations is a major concern for prolonged manned space missions, particularly those outside of the earth’s magnetic field. And because HZE particles are now being used for radiation therapy it becomes more important to understand the biological consequences of such exposures. There are currently five heavy particle treatment centers that utilize ^12^C with a number of other facilities under construction or being planned. Approximately 80,000 individuals have now been treated for various cancers with ^12^C with other ions under consideration. Given the differences in energy deposition patterns as the LET increases, there are likely differences in molecular response leading to different outcomes. And while there may be an enhancement in tumor control, for the normal tissue in the radiation field or an astronaut exposed to the GCR there may be an enhanced mutation frequency or enhanced genomic instability with the likelihood for increased risk for carcinogenesis. Therefore, characterization of the molecular response to HZE particle radiation is a key tool in the development of accurate risk assessment models for long-term space travel as well as the therapeutic use of HZE particles.

Prior risk models based upon epidemiology from predominantly the atomic bomb survivor data has determined that radiation-induced lung cancer has the greatest carcinogenic risk for solid tumors and this may be due to the number of cells at risk in the lung compared to other organs [17]. In prior work it was demonstrated that normal human bronchial epithelial cells (HBECs) could be immortalized with a combination of hTERT and CDK4 expression [18]. These cells cloned with high efficiency, differentiated into a ciliated epithelium under the right conditions, did not clone in soft agar, did not form tumors in immune-compromised mice, but could be genetically manipulated. We have recently reported on their response to γ-ray radiation with and without oncogenic manipulation [19,20]. In addition, with appropriate oncogenic changes, these HBECs can be progressed to full malignancy [21]. Thus, they make an excellent preclinical model for examining the molecular effects of HZE particle radiations and subsequent progression towards malignancy.

The HEBC3KT cell line, one of a series of normal human bronchial epithelial cell lines [18], was used as the sentinel cell line to determine the cellular and molecular radioresponse of our HBEC panel to radiations of increasing LET including γ-rays, and beams of the HZE particles ^28^Si and ^56^Fe both with nominal energies of 1 GeV/n (Giga electron volt per nucleon). Historically, experiments with a variety of cell lines have shown a large difference in cell killing and cellular transformation with exposures to particles of increasing Z and energy. We postulated that there were likely to be differences in the initial molecular responses of cells to such exposures that may drive the differences in cellular response. Experiments examining the long term changes in molecular signaling are ongoing. Here, besides cellular survival, we examined whole genome gene expression analysis over 24 h to determine whether the molecular responses in signaling were unique to LET. Our data indicated that while there is a strong similarity in the expression of particular radiation responsive signal transduction pathways there are also distinct gene expression profiles that were induced specifically by each radiation. A 73 gene signature was developed that segregated cells based upon the radiation exposed which was validated in blinded RNA samples isolated from cells exposed to γ-ray, ^28^Si or ^56^Fe.

## Methods

### Cell line and radiation

The human bronchial epithelial cell line HEBC3KT was derived at UT Southwestern Medical Center. HEBC3KT was first isolated from normal bronchial epithelial tissue and then non-oncogenically immortalized through the overexpression of CDK4 and hTERT. The cells have been shown to have a normal HBEC phenotype and normal p53 checkpoint function after radiation [18]. Cells were grown in a 95% air, 5% CO_2_ environment at 37°C in Keratinocyte Serum Free (KSF) medium supplemented with epithelial growth factor and bovine pituitary hormone (Gibco, Life Technologies, Grand Island, NY USA).

High energy HZE particle radiations were carried out at the NASA Space Radiation Laboratory (NSRL), at Brookhaven National Laboratory while the γ-ray exposures were conducted using a ^137^Cs source at the Department of Biology, Brookhaven National Laboratory. Cells were irradiated at 60-80% confluence as determined by eye. Experiments were repeated three times during 5 separate campaigns within a two-year period. The species of ions, kinetic energy and LETs are listed in Table 1.

**Table 1:** Experimental design using different radiation types, doses and time points

**Radiation**	**Energy (MeV/n)**	**LET (KeV/μm)**	**Dose (Gy)**	**Post IR Time (hour)**	**Exp date**
^56^Fe	1000	150	0, 0.5, 1	0, 1, 4, 12, 24	Mar. 2008
					Nov. 2008
					Nov. 2009
^28^Si	1000	44	0, 0.5, 1	0, 1, 4, 12, 24	Apr. 2009
					May. 2009
					Nov. 2009
γ-ray	0.662	0.2	0, 1, 3	0, 1, 4, 12, 24	Nov. 2008
					Apr. 2009
					Nov. 2009

### Clonogenic assays

Relative cell survival was determined as follows. Four hours after irradiation, cells were trypsinized, counted, and plated into 100 mm dish. Both control and irradiated cells were cultured for 9-14 days, stained with 0.5% crystal violet in 1% formalin-PBS, and counted for colonies of more than 50 cells. Survival curves were plotted using a two-component survival fit. The Relative Biological Effectiveness (RBE) was calculated based on equations of the fit curve. Clonogenic assays were performed over at least two different NSRL campaigns with each experiment performed in triplicate. The error bars represent standard error.

### RNA labeling and microarray hybridization

Cells were trypsinized, collected as a pellet of cells by micro-centrifugation at 10,000 rpm and flash-frozen with dry ice at 1, 4, 12 and 24 hours after each radiation. At each time point, a mock cell sample without radiation was also collected as a cell pellet and flash-frozen. An extra mock sample before radiation as common reference was also collected. Total RNA isolation was conducted later at the time of microarray analysis using Qiagen RNeasy Mini Kit according to manufacturer’s manual.

Illumina HumanWG-6 V2 BeadChip (Illumina, Inc.) human whole-genome expression arrays, which contain 48,701 probes on each array, were used in this study. Each RNA sample was amplified by Ambion TotalPrep RNA amplification kit with biotin UTP (Enzo) labeling, using 500 ng of total RNA. The Ambion Illumina RNA amplification kit uses T7 oligo(dT) primer to generate single stranded cDNA followed by a second strand synthesis to generate double stranded cDNA which is then column purified. In vitro transcription with T7 RNA polymerase generated biotin-labeled cRNA. The cRNA was then column purified, checked for size and yield using the Bio-Rad Experion system, And then 1.5 μg of cRNA was hybridized to each array using standard Illumina protocols with streptavidin-Cy3 (Amersham) being used for detection. Slides were scanned and fluorescence intensity captured using an Illumina BeadStation.

### Data processing and visualization

Expression values from 134 expression arrays of Illumina HumanWG-6 V2 BeadChip were extracted using BeadStudio v3.3. The data was background subtracted and quantile-normalized using the MBCB algorithm [22-24]. Expression values for each sample were then normalized to the signal from the reference sample before radiation for each radiation type and for each different experiment. To discern sample signal variations for subsequent analysis by hierarchical clustering or principal component analysis (PCA), the sample signal was further normalized to that of the mock irradiated samples taken at each time point. The data then underwent batch correction using Partek Genomics Suite software (version 6.5) to correct for any batch effect created by the different NSRL campaigns. Averaged log2 ratios were used for analysis. The hierarchical clustering was performed using Spearman absolute value dissimilarity metrics and Ward’s method was used as the clustering method. The PCA was performed using a covariance matrix and method of eigenvector scaling was normalized. Clustering and PCA analysis were implemented using Partek Genomics Suite version 6.5. The whole set of microarray data were also deposited in the GEO database, accession number GSE44282.

### Significance, gene function and pathway analysis

Significance analysis to find differentially expressed genes was performed using the maSigPro package from Bioconductor [25]. To increase statistical power, genes with Illumina Detection P-values > 0.05 were excluded in all sample sets. These genes typically had low base-line expression and were considered as background signal. The significance cutoff used was a False Discovery Rate (FDR) < 0.01 corrected for multiple testing using the Benjamini and Hochberg correction and an R-squared threshold of 0.3. For each radiation type, the common genes from the two radiation doses were subjected to fold change cutoff (> 1.3). Gene function and pathway analysis was performed using Ingenuity Pathway Analysis software.

### Prediction analysis of radiation qualities

Prediction analysis was performed using the Partek Genomics Suite version 6.5. A compiled gene list was created using a more stringent R-squared cutoff (> 0.5) from significance analysis. Genes specifically changed by different radiation qualities were combined and filtered for probes that were well annotated in the RefSeq database. Log2 ratios of irradiated samples normalized to reference RNAs before radiation were used in the prediction study. Classification methods were tested using K-Nearest Neighbor, Nearest Centroid and Support Vector Machine, Diagonal Discriminant Analysis and Partial Least Squares. Model selection and accuracy estimation were performed using a 2-level cross-validation strategy available from Partek. Briefly, several iterations of model validation and testing procedures were executed. With each iteration, one fourth of the samples were randomly selected as the testing set. The remaining samples were divided into 3 parts and each part was again partitioned into 10, with 9/10 for model training and 1/10 as validation. The best model was used for the testing set to calculate accuracy rate. The classification algorithm and parameters were as follows: K-nearest neighbor, number of neighbor candidates were 1,3 and 5; Nearest centroid, prior probabilities were equal and proportional; Diagonal discriminant analysis, function and prior probabilities were linear with equal probability; Support Vector Machine, kernel was polynomial, cost was 1 to 1001 and step was 100, tolerance was from 0.001 to 0.001 step 0.01; Partial least squares, function and prior probabilities were linear with equal probability. The average accuracy for all iterations was used as the final accuracy rate. The model was deployed to predict the radiation type from a blinded dataset generated by independent radiation exposures carried out some 6 months after the prediction set was developed.

### Real time quantitative RT-PCR

Complementary DNA was synthesized from the treated RNA solution in a reaction containing SuperScript III reverse transcriptase (Invitrogen) and random hexamer primers. The gene specific primers were designed by using Primer3 software. PCR reactions were performed using a SYBR PCR master kit (AB Biosystems, Inc.), and a Chromo4 Fluorescence Detector (Bio-Rad, Inc.). The PCR protocol was designed with an initial denaturing step of 95°C 10 minutes, followed by 40 cycles of 95°C 15 seconds and 60°C 1 minute. Serial dilutions of cDNA synthesized from human reference RNA (Stratagene, Ivc.) were used to describe standard curves for each gene. Human GAPDH was used as an internal control between samples. The PCR reactions were performed in triplicate for each gene being validated. Primers used in this experiment can be found in the Additional file 1. Data analysis was performed using Opticon software from Bio-Rad. The amount of RNA was normalized with the internal control and the ratios were generated using irradiated samples divided by mock samples.

## Results

### Relative biological effectiveness of cytotoxicity after HZE radiation in normal bronchial epithelial cells

**Figure 1:**
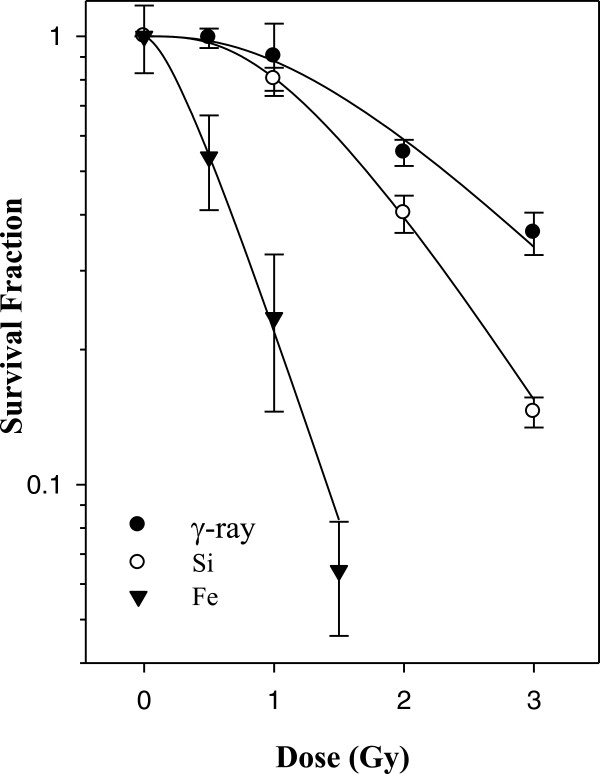
**Clonogenic survival of HEBC3KT cells.** Clonogenic survival of HEBC3KT cells were irradiated by ^56^Fe, ^28^Si or γ-rays. Survival curves were plotted using a two-component survival fit. The data represents two radiation experiments for each dose and radiation type. Error bars: standard error.

Clonogenic assays performed on HEBC3KT cells after ^56^Fe, ^28^Si and γ-ray irradiation indicated that Fe ion irradiation resulted in the most severe cell killing, while ^28^Si irradiation induced moderate cell killing and the survival curve falls between γ-ray and Fe radiation (Figure 1). The highest dose in this survival study was 3 Gy for γ-ray radiation and resulted in 34% of cell survival according to the fit curve. At this cell survival level the relative biological effectiveness (RBE) for ^56^Fe radiation was 3.91 while the RBE for ^28^Si was 1.38. Irradiation at 1 Gy for γ-ray resulted in 88% cell survival with RBEs of 5.92 and 1.23 for ^56^Fe and ^28^S, respectively. Radiation doses for subsequent gene expression studies were chosen based on the cell killing data so as to cover the range of RBEs between ^56^Fe/γ-ray and ^28^Si/γ-ray, such that we can compare gene expression differences at the same doses as well as at or near the same biological effect, that is, cell killing.

### Distinct gene expression profiles in HBECs in response to different radiation qualities

**Figure 2:**
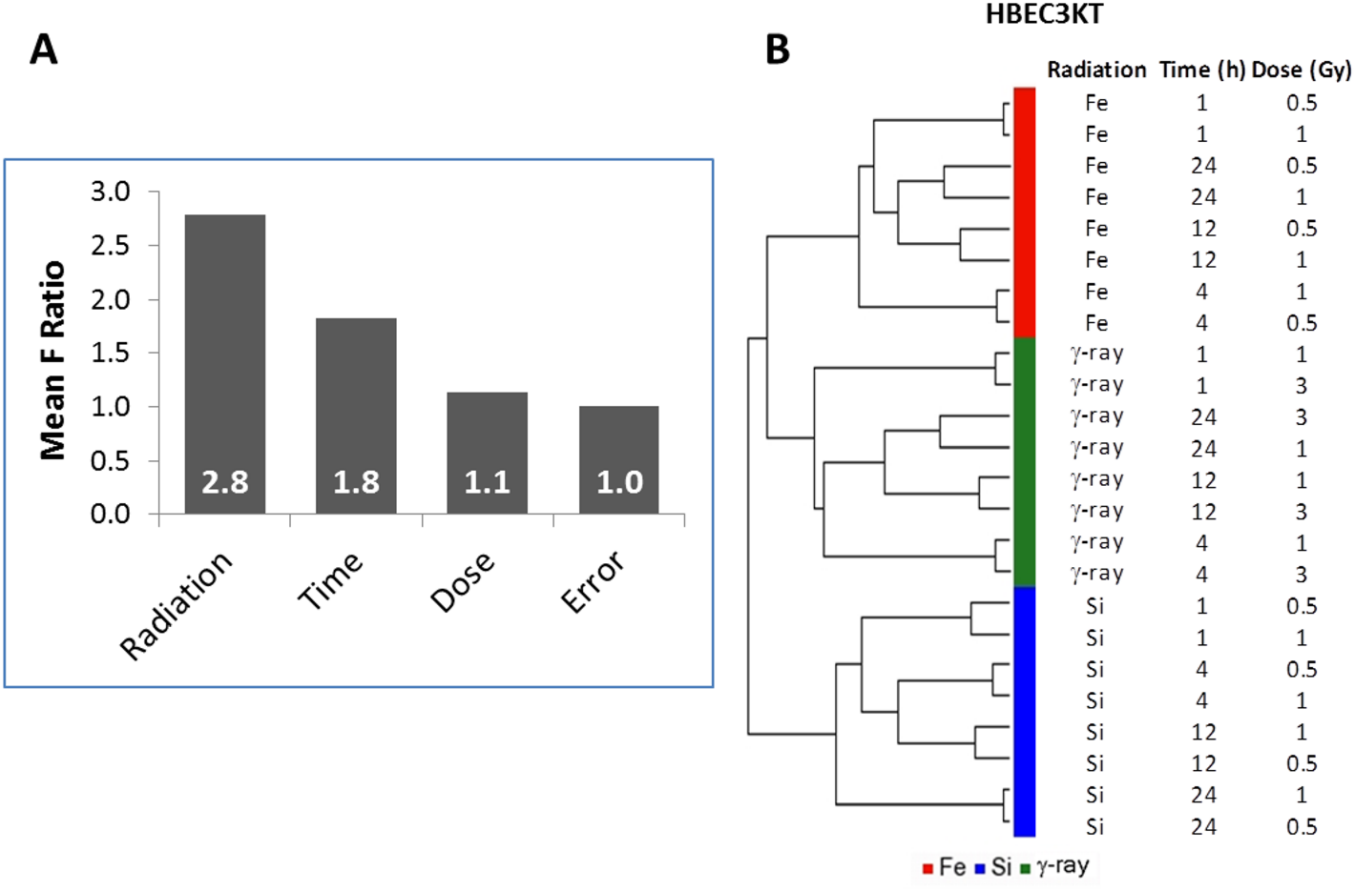
**Factor and unsupervised cluster analysis of transcriptome profiles.** Radiation quality was the major source of variation in the model of differential gene expression profiles in response to HZE and γ-ray radiation in normal HBEC cells. **A**, Sources of variation obtained from ANOVA suggested that radiation quality and post-radiation time were the major sources of variation. Error indicated the random noise level of the ANOVA model. **B**, Unsupervised hierarchical clustering of gene expression profiles that were normalized to mock-irradiated controls suggest distinct responses of cells to different radiation types.

An ANOVA model was developed in an attempt to reveal which experimental factors in this study contributed the most to gene expression changes. The ANOVA model included 3 factors; radiation type, dose and time post-irradiation. Average F ratios were calculated as the mean of F ratios of all genes for each factor (Figure 2). The results indicated that radiation type was the most significant source of variation in overall gene expression (Figure 2A). Post-IR time had a more modest contribution while dose was at the noise level. Unsupervised sample clustering aligned with experimental conditions and was consistent with the factor analysis. Normal epithelial cells (HEBC3KT) were clustered into 3 major groups according to radiation type (Figure 2B). Different doses were paired precisely at each time point, indicating that the kinetics of gene expression for different doses were similar. A detailed clustering analysis with a heatmap generated from log2 ratios of 48,701 probes is found in the Additional file 2. The overall results suggested that different radiation types induced distinct gene expression profiles. The effect from different doses, irrespective of the extent of cell survival was not significant enough to change the profiles specific to different radiation types. Accordingly, we treated different doses as replicates in our subsequent analysis of gene expression profiling.

### Significance analysis indicates that unique responses to radiation qualities are more prominent than common responses

**Figure 3:**
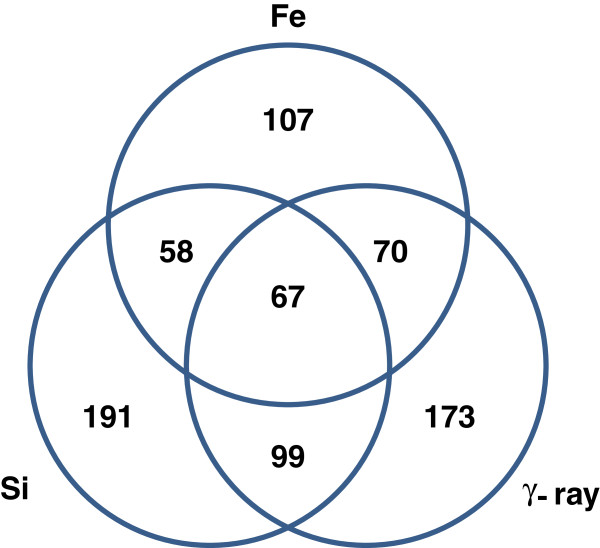
**Differentially expressed genes in response to **^**56**^**Fe, **^**28**^**Si and γ-ray irradiations.** Venn diagram showed the numbers of genes, either specific to or overlapping with different radiation types.

**Figure 4:**
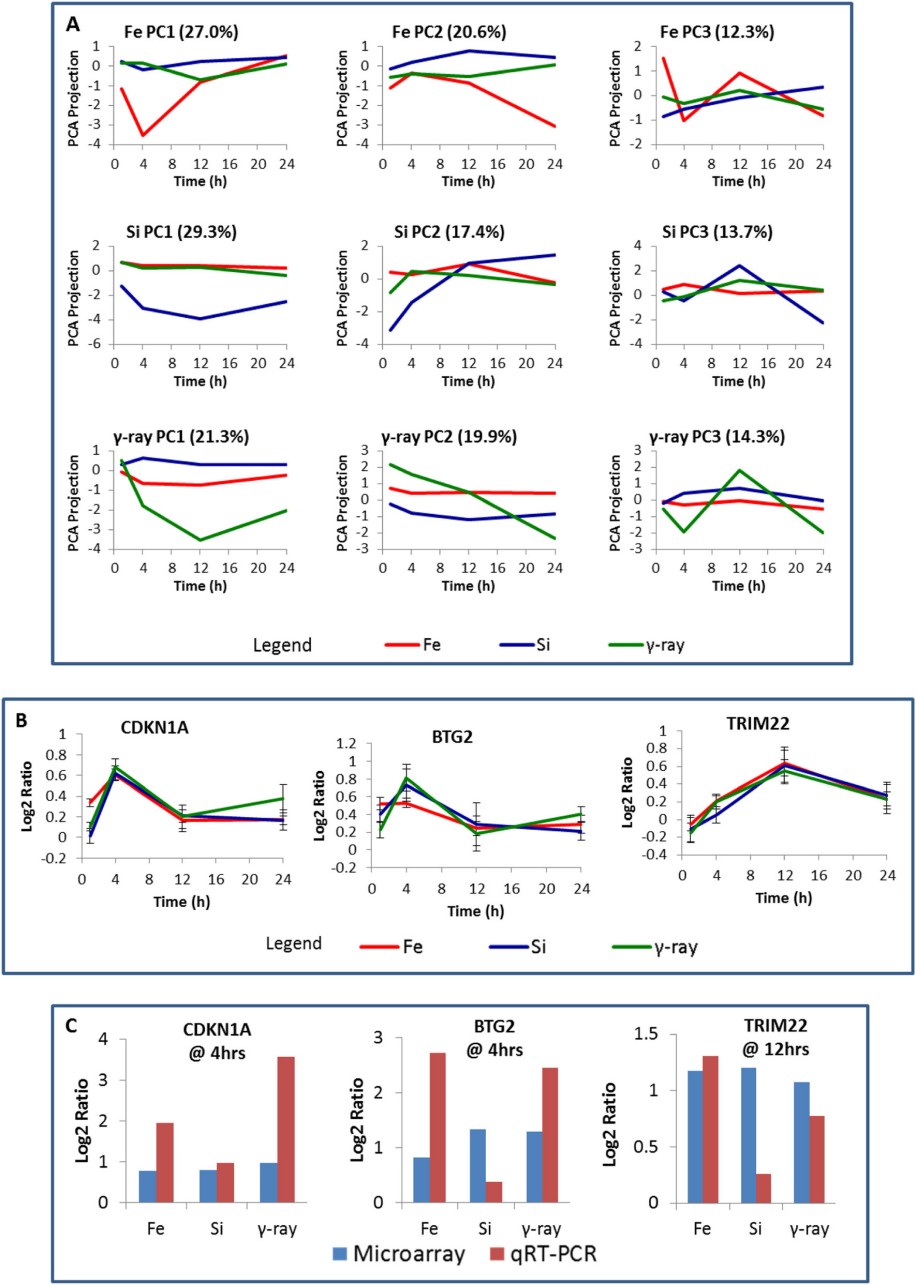
**Temporal patterns of significantly changed genes in response to **^**56**^**Fe, **^**28**^**Si and γ-ray irradiations. A**, The major temporal patterns of genes whose expression is associated with a particular radiation type are displayed. These patterns correspond to the first three components from principal component analysis (PCA). **B**, Genes associated with p53 signaling and/or involved in cell cycle regulation exhibited similar kinetics of expression in response to different radiation qualities. Average log2 ratios from three replicate experiments were used in the plot. Error bar: standard error. **C**, Using a single representative experiment, microarray expression values at a given time for the genes in Figure 4B were validated using qRT-PCR. The log2-ratios of irradiated *vs*. mock samples from both qRT-PCR and microarray analysis of the corresponding replicate were plotted. The up-regulation of these genes at the indicated time points was confirmed.

Significance analysis of gene expression changes of normal epithelial cells (HEBC3KT) over the 24 hour time period was performed using the maSigPro package in Bioconductor. Differentially expressed genes with statistical significance were determined by a 2-step regression analysis in the normal HEBC3KT cells with FDR < 0.01 and fold change cutoff of greater than 1.3. In total 765 genes were selected as significantly changed, exhibiting different expression kinetics in comparison with control groups. Significant differences in response to different radiation qualities were demonstrated with large groups of differentially expressed genes that were specific to certain radiation types. There were 173 genes that were significantly changed only after γ-ray radiation, 191 genes were changed only after ^28^Si radiation and 107 genes were changed only after ^56^Fe radiation (Figure 3). Major temporal expression patterns of the 3 groups of genes were analyzed by PCA. The top 3 principal components represented over 50% of genes in each radiation group (Figure 4A). A relatively small number of differentially expressed genes overlapped across the three radiation types (Figure 3). Most of the overlapping genes showed different temporal patterns for different radiation types. Only 7 genes, *CDKN1A*, *TRIM22* and *INPP5D*, *BTG2*, *C7orf10*, *GLUL* and *CCNA1*, showed similar temporal patterns in all radiation types. Three out of the 7 genes, *CDKN1A*, *BTG2 and TRIM22*, are direct p53 targets and/or involved in cell cycle inhibition (Figure 4B). From the three experiments, a single time from a single experiment was selected in order to validate the microarray results by quantitative RT-PCR (Figure 4C). The time chosen was that of maximum differential expression for that gene as indicated in Figure 4B. And while the specific values for log2 ratios were not always equivalent, the log2-ratios of irradiated *vs*. control samples were greater than 0 for both the qRT-PCR and the microarray data, indicating consistent up-regulation for these genes (Figure 4C).

### Gene signatures predict radiation quality with high accuracy

A gene list was generated by significance analysis using a more stringent R-squared value cutoff (> 0.5) in order to obtain a smaller gene list which was highly specific to each radiation type and that would avoid over-fitting of the prediction models with a large number of genes. Probes with ambiguous annotations such as predicted genes or EST sequences were removed. The final list of 73 genes with different expression patterns was used to build models to predict radiation quality in 84 irradiated samples of HEBC3KT cells. The overall accuracy of prediction was estimated using a Partek algorithm that used a 2-level nested cross validation strategy. Five classifiers were tested including Nearest Centroid (NC), K-Nearest Neighbor (KNN), Support Vector Machine (SVM), Diagonal Discriminant Analysis (DDA) and Partial Least Squares (PLS). The results showed high accuracies for all classifiers (Table 2). Then, 24 samples irradiated in the Fall 2009 campaign to be used as a blinded dataset, were collected. The SVM model was used to test this dataset and it predicted radiation qualities for 23 of the 24 samples, an accuracy of 96%. A detailed list of the 73 gene signature was provided in Additional file 3.

**Table 2:** Prediction accuracies by radiation type using different gene classifiers

**Classifier**	**Class**	**Samples per class**	**# Correct**	**# Errors**	**% Correct**	**% Error**
NC	^28^Si	24	23	1	94	6
	γ-ray	24	22	2	90	10
	^56^Fe	36	36	0	100	0
	**Total**	84	80	4	**95**	5
KNN	^28^Si	24	22	2	92	8
	γ-ray	24	23	1	96	4
	^56^Fe	36	36	0	100	0
	**Total**	84	81	3	**96**	4
SVM	^28^Si	24	23	1	96	4
	γ-ray	24	24	0	100	0
	^56^Fe	36	36	0	100	0
	**Total**	84	83	1	**99**	1
DDA	^28^Si	24	23	1	96	4
	γ-ray	24	23	1	96	4
	^56^Fe	36	36	0	100	0
	**Total**	84	82	2	**98**	2
PLS	^28^Si	24	20	4	83	17
	γ-ray	24	18	6	75	25
	^56^Fe	36	32	4	89	11
	**Total**	84	70	5	**83**	17

*NC* Nearest Centroid, *KNN* K-Nearest Neighbor, *SVM* Support Vector Machine, *DDA* Diagonal Discriminant Analysis, *PLS* Partial Least Squares.

### Gene function and pathway analysis reveals differences and commonalities in response to radiation quality

**Figure 5:**
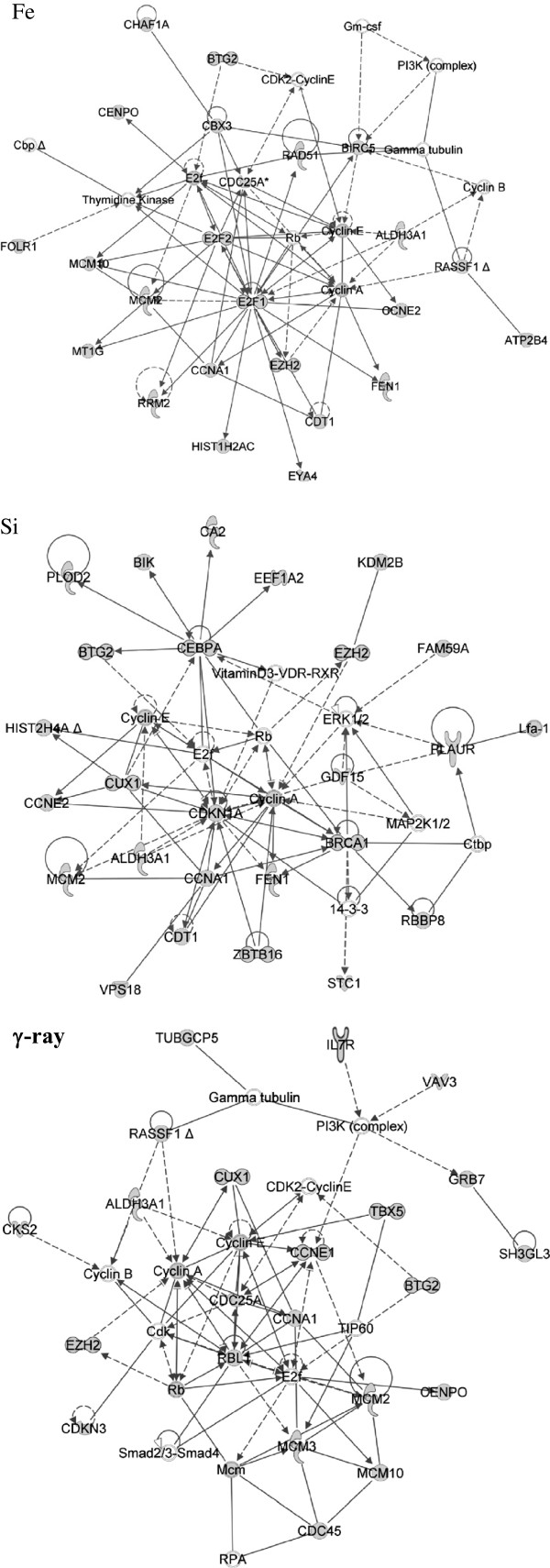
**Gene interaction networks associated with radiation types.** Highest rated gene interaction networks for differentially expressed genes after exposure to ^56^Fe or ^28^Si particles or γ-rays. Nodes with grey color identify significantly changed genes after radiation.

**Figure 6:**
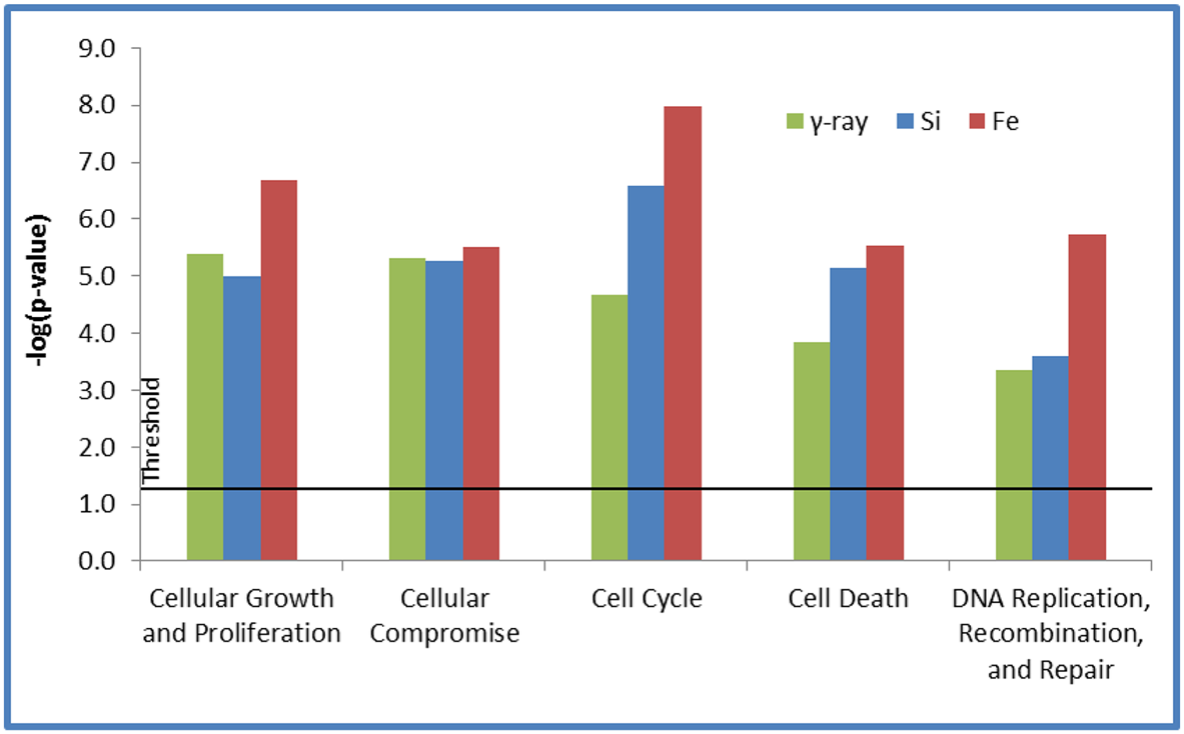
**Gene function analysis for differentially expressed genes after radiation.** Gene enrichment analysis using Ingenuity pathways Analysis software for identification of common gene function categories regardless of radiation type. The horizontal line denotes a significance level of (p<0.05).

**Figure 7:**
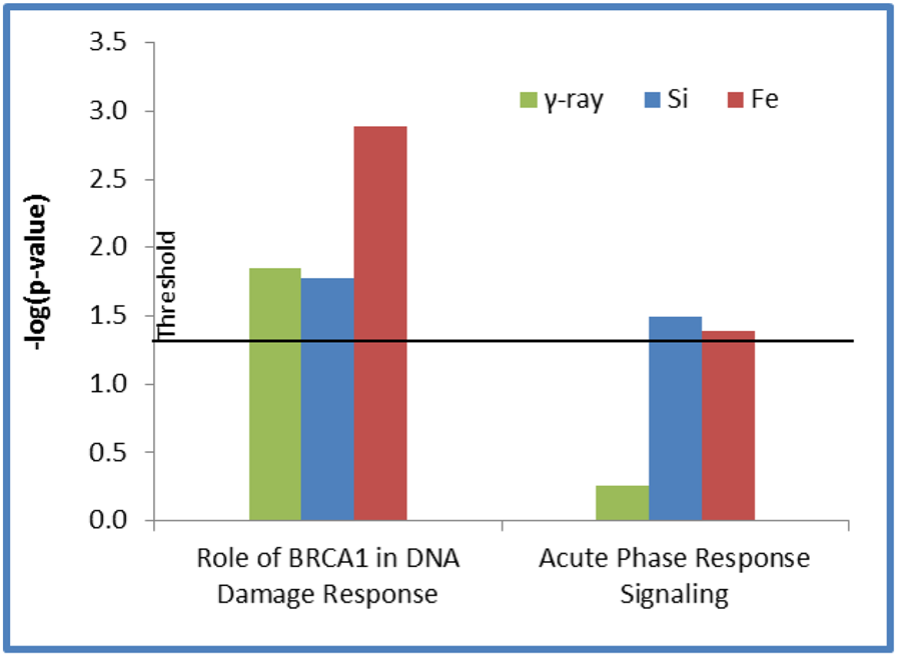
**Signal transduction pathway analysis of differentially expressed genes after radiation.** Gene enrichment analysis identified the BRCA1-involving DNA damage response pathway responded after exposure to all 3 radiation types, whereas acute phase response signaling was more significant in response to HZE irradiation. The analysis was done using Ingenuity Pathway Analysis software, and the horizontal line indicates a significance level (p<0.05).

Using Ingenuity Pathway Analysis software, molecular functions and pathways for differentially expressed genes after radiation in normal HEBC3KT cells were examined. The highest ranked gene interaction networks developed from the list of genes that commonly responded to different radiation types were Cell Cycle; and DNA Replication, Recombination and Repair (Figure 5). Enrichment analysis of differentially expressed genes showed overlap of molecular function groups in response to all three radiation qualities, including Cell Cycle, Cell Death, DNA Replication, Recombination and Repair, Cellular Compromise, and Cellular Growth and Proliferation. The statistical significance cutoff for enrichment analysis was p<0.05 (Figure 6). Signaling pathway analysis showed that a *BRCA1*-centric DNA damage response pathway was also significantly activated (p < 0.05) in all 3 radiation types (Figure 7). Genes involved in this pathway included *CDKN1A*, *RBBP8*, and *RAD51*. The Acute Phase Response pathway was more significantly activated in HZE irradiation, with more than twice as many genes overrepresented in ^56^Fe and ^28^Si irradiated samples than in γ-ray irradiated samples (Figure 7). Several pathway changes were specific to certain radiation types. This was determined from genes involved in a particular pathway if they were only significantly changed after one type of radiation and none of the genes responded to the other two radiation types. Genes in Inhibition of Angiogenesis by *TSP1* and Mechanism of Viral Exit from Host Cells pathways only responded to γ-ray irradiation, whereas Notch signaling was specific to ^56^Fe and Phospholipase C signaling was specific to ^28^Si irradiation (Table 3). Overall, there is a commonality of gene function groups but differences in signaling pathways in response to different radiation types.

**Table 3:** Genes that activate unique pathways specific to radiation types in normal HEBC3KT

**Gene**	**Adjusted p-value**	**Radiation**	**Fold change**				**Specific radiation quality**	**IPA unique pathway**	**Gene interaction**
			**1 hr**	**4 hr**	**12 hr**	**24 hr**			
THBS1	0.0005	γ-ray	−1.1	1.0	1.1	**1.4**	γ-ray	Inhibition of angiogenesis by TSP1	Phosphorylation of CD36 and FYN, subsequently activates p38, p53 and caspase 3 to promote apoptosis.
	>0.01	Si	1.1	1.2	1.2	−1.1			
	>0.01	Fe	1.1	1.2	−1.1	−1.0			
SH3GL3	0.0001	γ-ray	**−1.3**	1.1	−1.1	1.2	γ-ray	Mechanism of viral exit from host cells	Phosphorylation of ALIX, whose activation blocks apoptosis
	>0.01	Si	1.2	−1.0	1.3	1.2			
	>0.01	Fe	1.0	−1.2	−1.1	1.0			
APH1B	>0.01	γ-ray	1.2	1.1	1.3	−1.0	Fe	Notch signaling	Cleaves Notch C-terminal fragment, release the active form NCID and translocate to nucleus
	>0.01	Si	1.0	1.2	−1.1	1.2			
	0.00002	Fe	1.0	**−1.3**	1.1	1.1			
BLNK	>0.01	γ-ray	−1.2	1.1	−1.2	1.2	Si	Phospholipase C signaling	BLNK, PLD1 and PLD3 are upstream molecules that activate PLC-β and PLC-γ. PLC-γ subsequently activates PKC and the latter activates ERK1/2 and NF-κB pathways.
	0.00002	Si	−1.3	1.2	1.1	**1.5**			
	>0.01	Fe	−1.0	1.1	1.1	1.1			
PLD1	>0.01	γ-ray	−1.0	−1.2	−1.2	1.2	Si		
	0.0002	Si	−1.2	1.1	**1.4**	−1.0			
	>0.01	Fe	1.0	1.2	1.1	−1.1			
PLD3	>0.01	γ-ray	−1.2	−1.1	1.2	1.0	Si		
	0.0002	Si	−1.1	1.2	1.3	**1.6**			
	>0.01	Fe	−1.2	−1.2	1.1	−1.3				

## Discussion

Although it is well known that the biological effectiveness of ionizing radiation for cell killing, mutagenesis and carcinogenesis can differ with the type of radiation exposure, whether the molecular mechanisms for the different responses are novel is not straightforward. A number of researchers have suggested that the molecular response to very low doses may be different from that at higher doses [26,27] and that the response to very high, ablative, doses of ionizing radiation may be also be unique. Whether there are unique responses based upon particle quality was also unknown. Therefore, global transcriptome changes in HEBC3KT at three LETs, the very low LET of γ-rays (0.2 keV/μm), an intermediate LET (40 keV/μm) of 1 GeV/n ^28^Si, and that of 1GeV/n ^56^Fe (150 keV/μm), an LET that is at or near the maximum relative biological effectiveness for a number of endpoints were examined in order to provide a comprehensive picture of cellular responses at the molecular level.

Given the excess risk modeled for lung cancer for HZE particle exposure the HEBC3KT cell line, a non-oncogenically immortalized cell line which, unlike cells immortalized using viral oncoproteins, does not form tumors in immune-compromised mice was used. The HEBC3KT cells have also been shown to have an intact p53 checkpoint pathway in response to UV radiation [18]. These data also demonstrated consistent over-expression of p53 target genes such as CDKN1A and BTG2, after ionizing radiation, suggesting that the cell line is a good model to study the initial molecular events in normal epithelial cells after exposure to different radiation types. As others have reported, cell survival was LET dependent. The range of RBEs for ^56^Fe was from 3.91 to 5.92, the range for ^28^Si irradiation was from 1.38 to 1.23, depending on different cell survival levels, *i*.*e*., 33.8% or 88.1% of cell survival in our assays. The range of radiation doses chosen for expression studies encompassed the RBEs of each HZE particle so that we could study the dose effect at a similar survival level. The data suggested that the doses within this range were not a significant factor by which transcriptome profiles could be differentiated.

At the individual gene level significant differences in expression between the different radiation types were observed. Of the 765 genes that were differentially expressed after radiation, only 7 displayed a similar response to all radiation qualities across the time course. The differences were also evident by the sources of variation (SOV) obtained from ANOVA modeling, which indicated that radiation quality was the major contributor to the variations among other factors such as time after radiation and dose. A previous study has shown a dose dependent response after low-and high LET radiations in human mesenchymal stem cells [17]. In this data set, ANOVA SOV analysis showed that the dose effects were very small and similar to the level of background noise. This may be due to the differences in cell types and the intrinsic variation of experiments. Despite the variations of gene expression patterns, the reproducibility of the dataset was confirmed by highly consistent patterns of several genes involving p53-dependent cell cycle and cell death regulation in response to all radiation qualities. Amongst these genes, three were direct targets of p53. *CDKN1A*/*p21* is a p53-dependent kinase inhibitor that regulates G1/S and G2/M cell cycle check point. Over-expression of *CDKN1A*/*p21* mRNA was observed after γ-ray radiation in a p53-dependent manner. *CDKN1A*/*p21* has been demonstrated to be up-regulated with similar temporal patterns after low-LET radiation [26,28]. *CDKN1A*/*p21* has been shown over-expressed after ^56^Fe particle radiation in human skin fibroblast and lens epithelial cells [29,30]. While over 90% of the differentially expressed genes were different, these data indicated that *CDKN1A*/*p21* was over-expressed with the same temporal patterns after γ-ray, ^56^Fe and ^28^Si particle radiation, suggesting the essential role of p53-dependent p21 cell cycle check point control in response to ionizing radiation. *BTG2* is involved in the regulation of the G1/S transition of the cell cycle and has been confirmed as a p53-target. It is up-regulated after low-LET radiation exposures. *TRIM22*, or tripartite motif containing 22, is a p53 target and can be induced by interferon-γ. Over-expression of *TRIM22* has been shown to be anti-proliferative. Other genes that showed similar temporal patterns include *CCNA1*, *INPP5D*, *GLUL* and a gene with unknown function, *C7orf10*. *CCNA1*, or cyclin A1, binds to CDK2 and CDC2, regulating S-phase and G2 phase of the cell cycle. *INPP5D*, *inositol polyphosphate*-*5*-*phosphatase*, has been shown to be a negative regulator of cell proliferation. *GLUL*, glutamate-ammonia ligase, regulates the metabolism of glutamine which is a main source of energy and involved in cell signaling and proliferation.

The differences in expression profiles were supported by a high prediction accuracy for classification of radiation type. Starting with genes that specifically responded to different radiation qualities, the gene list was optimized by excluding poorly annotated probes according to the RefSeq Database. Five classification algorithms were tested and Support Vector Machine (SVM) demonstrated the best prediction accuracy. The overall accuracy estimation was made by doing 4 iterations of training and testing processes, each time about 1/4 of the samples were held for the testing set and the others were used as training and validation sets. On average, 83 samples were predicted correctly out of 84 samples across the two-year experimental period. The accuracy rate was 99% when a SVM classifier was used. The SVM model was further deployed to test a blinded dataset collected 6 months later. The model successfully predicted radiation types for 23 out of 24 samples, a prediction accuracy of 96%. It has been shown that radiation response is specific to cell types from different tissue origins. A previous study identified the down-regulation of a set of histone genes in human lymphoblastoid cell lines 24 hours after equitoxic low- and high-LET radiations [31]. These changes were not observed in our model of HEBC3KT cells. There are likely a number of reasons for such differences, including but not limited to differences in cell type, i.e. lymphoid *vs*. epithelial, cell cycle time, cell cycle distribution at the time of irradiation or shifts in cell cycle distribution post-irradiation, perhaps even because of the mechanism of cell death. This suggests that the gene signature we developed is cell-type specific.

Gene function analysis revealed commonalities and contrast for individual gene expression changes. Top gene interaction networks consisted of different genes changed in response to different radiation qualities, however, all of the ontology analysis pointed to Cell Cycle, DNA Replication, Recombination and Repair pathways. These findings were consistent with known biological effects of HZE and γ-ray radiation which were related to cell cycle regulation and chromosome damage. Despite the commonality of interaction networks, most of the genes in theses pathways were either different, or they were same genes but responded with different kinetics. Detailed study is needed to decipher how these differences contributed to different biological effects associated with radiation qualities. Molecular signaling pathway analysis revealed more differences which were dependent on radiation type. Comparing low-LET γ-ray irradiation, HZE irradiation (^56^Fe and ^28^Si) induced more significant expression changes in the acute phase response pathway. This pro-inflammatory pathway may possibly explain the more severe biological effect induced by HZE particles or suggest a heightened risk for carcinogenesis in surviving cells. Several signaling pathways were shown as radiation quality-specific, *i*.*e*., gene members in the pathway only responded to one radiation quality. Two pathways, Inhibition of Angiogenesis by *TSP1* and Mechanism of Viral Exit from Host Cells, were specifically changed after γ-ray radiation while ^56^Fe and ^28^Si exposure did not cause any expression changes in these pathways. Two expression changes, up-regulation of *THBS1* and down-regulation of *SH3GL3*, occurred only after γ-ray irradiation. Both of the genes activate their downstream target by phosphorylation. Phosphorylation of *THBS1* targets results in pro-apoptosis signaling whereas phosphorylation of S*H3GL3* targets abrogates apoptosis signaling. The changes of these two genes after γ-ray irradiation were consistent with a pro-apoptosis signaling. ^56^Fe irradiation suppressed expression of *APH1B*, which is part of γ-secretase. This likely results in interference with Notch activation and translocation into the nucleus. ^28^Si irradiation specifically activated Phospholipase C Signaling by up-regulating expression of *BLNK*, *PLD1* and *PLD3* genes. These molecules activate *PLCγ*, which hydrolyzes *PIP2* and releases the second messenger *DAG*. The latter activates Protein Kinase C which participates in activation of stress responding and cell proliferation pathways such as NF-KB and ERK signaling.

## Conclusions

In summary, our data indicate that normal human bronchial epithelial cells exposed to HZE particle and γ-ray radiation elicit distinct gene expression patterns that are specific to the radiation type. Molecular functions involved in these distinct expression profiles were commonly associated with cell cycle regulation, DNA damage response and other stress responding mechanisms. Gene enrichment in specific pathways indicated that different molecular mechanisms are involved in the response to different radiation qualities. At least over the doses tested, dose does not appear to be a strong factor in eliciting differential transcriptome responses. What we have not discerned is how these differences in initial response are linked to the long-term consequences of such exposures. Experiments using long-term cell cultures from surviving cells are ongoing. Transcriptome data, measures of genomic instability, mutation within driver genes, cellular transformation and other endpoints are being collected. Ultimately, the fate of HBECs will be followed from the initial events following γ-ray and HZE particle exposures as described here, to the extent to which these irradiated cells form tumors in immune-compromised mice. Whether these initial differences remain over time or whether they coalesce into a common set of biomarkers or risk factors as cells undergo transformation and ultimately become oncogenic is currently unknown. Having such knowledge will be crucial for understanding: radiotherapy risks such as second cancers when using HZE particles for therapy; whether the novel pathways associated with only γ-ray exposures may be informative for development of biomarkers for determining risk for lung cancers in individuals who have or will receive multiple diagnostic CT scans over their lifetime [32-34]; and finally, the risk for lung cancers for astronauts on deep space or extended lunar missions.

## Competing interests

The authors declare that they have no competing interests.

## Authors’ contributions

LHD participated in designing the experiments, carried out irradiation, microarray assays, data analysis and quantitative RT-PCR. SP and MP participated in irradiation and sample preparation. LG helped setting up and maintaining a sample database. YX helped in data analysis. JDM helped to coordinate and draft the manuscript. MDS conceived of the study, participated in its design, coordination and helped to draft the manuscript. All authors read and approved the final manuscript.

## Supplementary Material

Additional file 1 Primers used for qRT-PCR. Click here for file

Additional file 2 Clustering Analysis of Gene Expression Profiles with Heatmap. Click here for file

Additional file 3 Ratio of differential expression of 73 gene signature that predicts Fe, Si and γ-ray irradiation. Click here for file
