# Supplementary material for: Distinct transcriptome profiles identified in normal human bronchial epithelial cells after exposure to γ-rays and different elemental particles of high Z and energy
Source: BMC Genomics. 2013 Jun 1;14:372. doi: 10.1186/1471-2164-14-372 (PMC3680091; doi:10.1186/1471-2164-14-372)
Supplement: Additional file 2 — Clustering Analysis of Gene Expression Profiles with Heatmap. [file 1471-2164-14-372-S2.pdf]

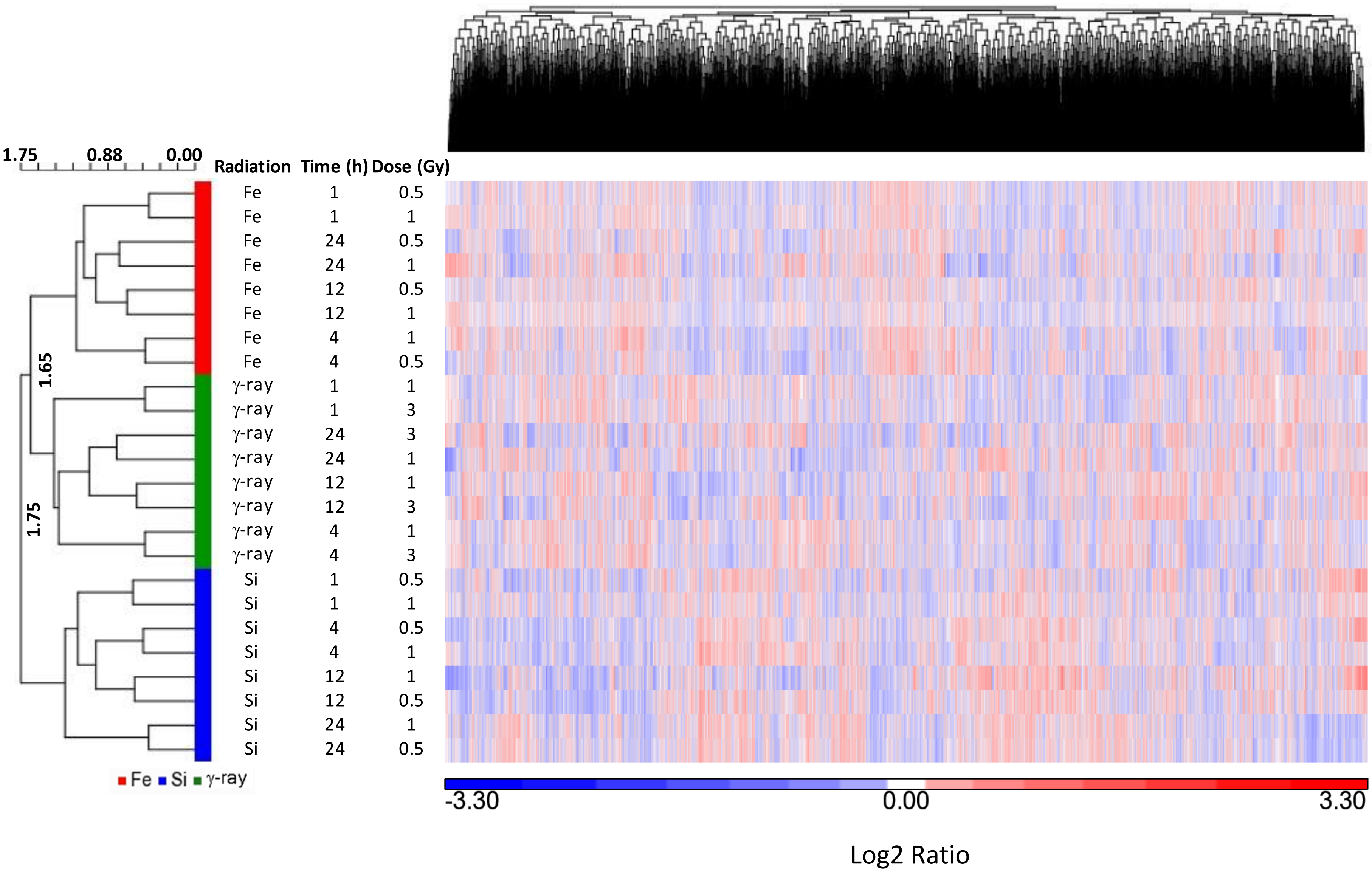


Unsupervised hierarchical clustering with a heatmap. The clustering was performed using averaged log2 ratios of 48,701 probes and implemented by Partek Genomics Suite 6.6. The row clustering was performed by using Spearman absolute value dissimilarity and Ward's method for grouping. The scale for the dendrogram represents the distance of clusters generated by Ward's method. The distances of clusters between 3 radiation types are labeled in the dendrogram. The column clustering was using Pearson dissimilarity and complete linkage.
